# NMR Metabolomics in Ionizing Radiation

**Published:** 2016-09-08

**Authors:** Jian Zhi Hu, Xiongjie Xiao, Mary Y Hu

**Affiliations:** Division of Earth and Biological Science, Pacific Northwest National Laboratory, USA

Ionizing radiation is an invisible threat that cannot be seen, touched or smelled and exists either as particles or waves. Particle radiation can take the form of alpha, beta or neutrons, as well as high energy space particle radiation such as high energy iron, carbon and proton radiation, etc [1]. Non-particle radiation includes gamma- and x-rays. Publically, there is a growing concern about the adverse health effects due to ionizing radiation mainly because of the following facts. (a) The X-ray *diagnostic images are* taken routinely on patients. Even though the overall dosage from a single X-ray image such as a chest X-rays scan or a CT scan, also called X-ray computed tomography (X-ray CT), is low, repeated usage can cause serious health consequences, in particular with the possibility of developing cancer [2,3]. (b) Human space exploration has gone beyond moon and is planning to send human to the orbit of Mars by the mid-2030s. And a landing on Mars will follow. (*"Obama Promises Renewed Space Program". The New York Times*. Retrieved April 15, 2010). Completely shield the high energy space radiation in outer space is a big challenging [4,5]. (c) The impact of past nuclear disasters such as Chernobyl disaster (1986/4/26) and Fukushima Daiichi nuclear disaster (2011/3/12) are long lasting, including leaving behand radiation contaminated sites that are very difficult to clean [6,7]. And (d) Radiological hazards are likely to be employed by terrorists *via* nuclear detonation, radiological dispersion devices, and covert placement/distribution of radioactive substances [8]. The worst case scenario for a radiation incident would involve a nuclear detonation-either from an improvised nuclear device or an actual warhead.

All cells can be damaged by ionizing radiation, but actively dividing cells are far more radiosensitive than cells that are neither meiotically nor mitotically active. The most radiosensitive cells in the human body include the bone marrow stem cells, gastrointestinal villi cells, and the gametes in the ovaries and testes. Acute Radiation Syndrome (ARS) is an illness caused by partial or whole-body exposure to high doses of ionizing radiation over a short period of time (usually a few minutes or less). According to American military radiologists, the pathophysiology effects dependence upon the irradiation doses are summarized in Table 1 [9]. Although the manifestations of radiation injury vary depending on total absorbed radiation dose and the preexisting health of the victim, it is clear from Table 1 that in most radiation scenarios, injury to the hematopoietic system and GI tract are the main determinants of survival. If left untreated, a victim exposed to a total dose of 3.5Gy (LD_50_ is about 4.0 Gy) and above is unlikely to survive.

The classical model of molecular injury involves immediate cellular damage following irradiation, which can result in membrane and intracellular injury, i.e, inflammation, DNA single and double strand break that subsequently turn on various genes and lead to cell proliferation, fibrosis, cancer or cell death [10–12]. Significant investigations at molecular level have been done at the genetic and protein levels by studying changes associated with DNA, RNA and proteins extracted from cells and animal tissues using genomic [13,14] and proteomic [15,16] methods. Although expensive and labor intensive, genomic and proteomic methods, may have potential as powerful tools for studying different levels of the biological response to radiation-induced injury, including searching for radiation specific molecular biomarkers. However, careful studies have generally shown a low correlation between the pattern of gene expression and the pattern of protein expression [17,18]. Moreover, even in combination, genomic and proteomic methods still do not provide the range of information needed for understanding integrated cellular function in a living system, since both ignore the dynamic metabolic status of the whole organism.

It is well-known that alterations in DNA, RNA and protein are associated with changes in metabolic profiles. Metabolites are chemical compounds that participate as reactants, intermediates, or byproducts in a cellular metabolic pathway, and include carbon compounds with a molecular weight typically in the range of 100–1000 Da. Radiation exposure will disturb the ratios and concentrations of endogenous metabolites, either by direct chemical reaction or by binding to key enzymes or nucleic acids that control metabolism. If these disturbances are of sufficient magnitude, toxic effects will result. Therefore, metabolomics, defined as a comprehensive and quantitative analysis of all metabolites in a biological system [19–21], will be an important new systems biology tool for elucidating the molecular mechanisms of radiation.

Metabolomics is a new technique and has only been recently applied in the field of radiation, emerging as a field of great significance for both translational and basic research [22–25]. Unlike approaches in which biomolecules/metabolites are selected and analyzed one or a few at a time, metabolomics focuses on broad identification and analysis of multiple metabolites simultaneously. The state of metabolome cumulatively reflects the stages of gene expression, protein expression, and the cellular environment as well as multidirectional interactions among these elements. Metabolomic information is complementary, yet distinct, from that generated by genomic and proteomic approaches. Moreover, metabolic changes are among the earliest cellular responses to environmental or physiological changes. It is well-known that there are estimated 30,000–40,000 genes (genome) associated with DNA, more than 100,000 transcripts (transcriptome) associated with RNA, and more than 1,000,000 proteins (proteome) yet there are only approximately 5000 metabolites (metabolome) in human cells [26,27]. It is clear that complexity is greatly simplified with metabolomics which, although in its infancy, has already proven capable of detecting and diagnosing a disease and evaluating the efficacy of therapy in an early stage [22,23,25,28]. Therefore, it is highly likely that metabolomics will provide valuable new information about the impact of radiation on human health.

Nuclear Magnetic Resonance (NMR) spectroscopy is a quantitative, non-destructive method that requires no or minimal sample preparation, and is one of the leading analytical tools for metabonomic research [19,29–33]. Unlike mass spectrometry based methods, where the peak intensity depends on the efficiency of ionization of the molecules that are different for different types of molecules and the ion suppression issues when multiple species coelute**,** the peak intensity in an NMR spectrum is directly proportional to the number or concentration of molecules. The easy quantification associated with NMR is a big advantage over other techniques. ^1^H NMR is especially attractive because protons are present in virtually all metabolites and its NMR sensitivity is high, enabling the simultaneous identification and monitoring of a wide range of low molecular weight metabolites, thus providing a biochemical fingerprint of an organism “without prejudice”. It is expected that NMR metabolomics will play an important role in understanding the damage at molecular level by ionizing radiation as have demonstrated recently by us [34,35].

Figure 1 shows an example [35] of applying ^1^H NMR metabolomics to study the changes in metabolic profile in the spleen of C57BL/6 mouse after 4 days whole body exposure to 3.0 Gy and 7.8 Gy gamma radiations. As an integrated part of NMR metabolomics, principal component analysis (PCA) [36], an unsupervised statistical method, and orthogonal projection to latent structures analysis (OPLS) [37], a supervised statistical method, are employed for classification and identification of potential biomarkers associated with gamma irradiation. The results from the PCA and OPLS analysis have shown [35] that the exposed groups can be well separated from the control group. Leucine, 2-aminobutyrate, valine, lactate, arginine, glutathione, 2-oxoglutarate, creatine, tyrosine, phenylalanine, π-methylhistidine, taurine, myoinositol, glycerol and uracil are significantly elevated while ADP is decreased significantly. These significantly changed metabolites are associated with multiple metabolic pathways and may be considered as potential biomarkers in the spleen exposed to gamma irradiation.

## Figures and Tables

**Figure 1:**
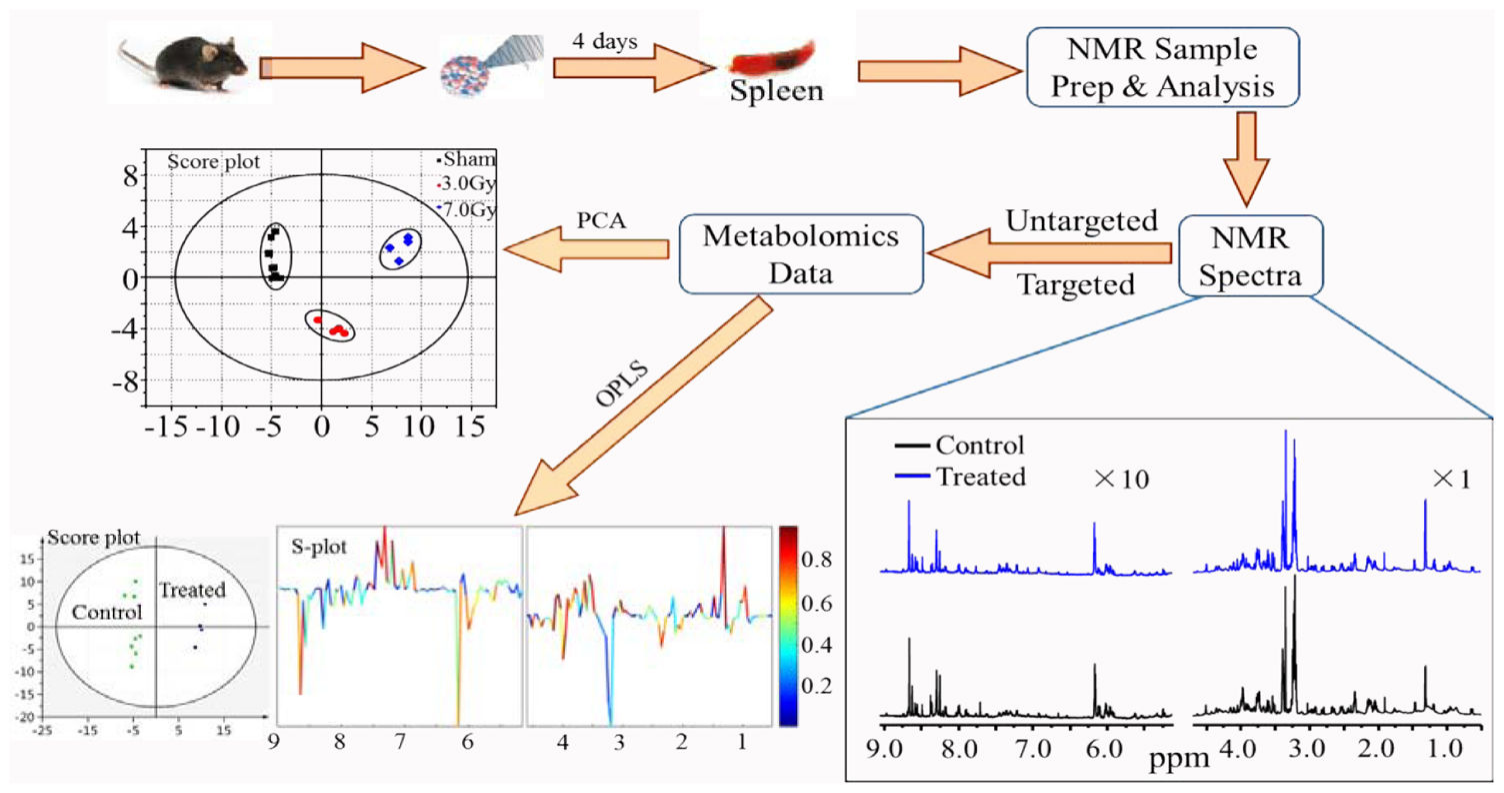
Example of applying ^1^H NMR metabolomics to study the changes in metabolic profile in the spleen of C57BL/6 mouse after 4 days whole body exposure to 3.0 Gy and 7.8 Gy gamma radiations.

**Table 1:** The phases of acute radiation syndrome and prognosis varying by dose.

Dose range (Gy)	Clinical Manifestations	Prognosis (Gy)(Untreated)
0.5–1.0	Slight decrease in blood cell count	Survival in vast majority
1.0–2.0	Early signs of bone marrow toxicity	Survival >90%
2.0–3.5	Moderate to Severe bone marrow toxicity	Survival likely
3.5–5.5	Severe bone marrow toxicity; GI damage mild	50% die within 3.5–6 weeks
5.5–7.5	Pancytopenia; Moderate GI damage	Death likely in 2–3 weeks
7.5–10	Severe Marrow & GI damage	Death likely within 1–2 weeks
10–20	Severe GI damage, radiation pneumonitis, altered level of consciousness, cognitive deficits	Death in 5–12days
20–30	Severe cerebro vascular dysfunction with hemodynamic collapse, fever	Death in 2–5days
